# Supplementary material for: User-Centered Design for Digital Patient-Navigation Tools in Oncology: Scoping Review
Source: JMIR Hum Factors. 2026 Apr 29;13:e87686. doi: 10.2196/87686 (PMC13173087; doi:10.2196/87686)
Supplement: Multimedia Appendix 2 [file humanfactors_v13i1e87686_app2.docx]

**Multimedia appendix.**

**Summary of Included Studies and Full Search Strategies**

**Table S1 – Summary Table Representing the Characteristics of Selected Studies**

|  | | | | | | | | | | |
| --- | --- | --- | --- | --- | --- | --- | --- | --- | --- | --- |
| **First author, year** | **Country Where the Study Was Conducted** | **Study Purpose** | **Cancer Context  (i.e., breast, colorectal, pediatric; or all cancers)** | **Target User Group   (i.e., patients, caregivers, navigators, clinicians)** | **Navigation Purpose   (i.e., Care coordination, Emotional/psychosocial support, Education/information provision, Appointment scheduling/tracking, Financial/insurance navigation, Patient-to-provider communication, Transportation/logistics, Overall care system navigation/access to care)** | **Type of Digital Modality (i.e., mobile app, web portal, EHR‑integrated portal, remote‑monitoring platform, telehealth/video, etc.)** | **UCD/HCD Phase  (i.e., discovery, prototyping, testing, implementation)** | **UCD/HCD Design Methods Used  (i.e., interviews, participatory sessions, journey mapping, co-design workshops, end-user feedback sessions)** | **Navigation Outcome(s) Reported   (i.e., Patient engagement, Usability, User satisfaction, Navigation effectiveness, Clinical utility, etc.)** | **Author’s conclusion** |
| ^9^ **Ngo, 2020** | USA | To understand the participants’ experiences with Personal Health Network (PHN) technology and to assess their perceptions of usability and usefulness of the PHN on care coordination during chemotherapy. | All (Adult) | Patients | Care coordination, Education/information provision, Appointment scheduling/tracking, Patient-to-provider communication | Mobile app | Testing | Interviews | Usability, User satisfaction, Navigation effectiveness | Care coordination is a valuable benefit for cancer patients undergoing chemotherapy, and the use of PHN technology can enhance this process by facilitating better communication and access to information. |
| ^12^ **Carr, 2023** | USA | To understand barriers affecting patient navigator workflows in breast cancer care and identify how a human-centered designed mHealth app could support navigators in improving patient care delivery. | Breast | Patients, Clinicians/Providers, Patient Navigators | Care coordination, Patient-to-provider communication, Overall care system navigation/access to care, Transportation/logistics | Mobile app | Discovery | Interviews, Content analysis | Navigation effectiveness, Feasibility and implementation logistics, Collaboration and coordination, Design and strategy insights | Stakeholder feedback offers critical insights into design elements for a mHealth app to support patient navigators in breast cancer care, with the study innovatively combining human-centered design and sociotechnical systems frameworks. |
| ^13^ **Duman-Lubberding, 2015** | Netherlands | To explore how health care professionals perceive follow-up care and the use of an eHealth tool in cancer follow-up. The application tracks patients' quality of life through patient-reported outcomes (PROs) and then provides automatic, personalized feedback and support recommendations. | Head and neck | Clinicians/Providers | Education/information provision, Overall care system navigation/access to care | Web platform | Discovery, Prototyping | Interviews | Usability, User satisfaction, Navigation effectiveness, Health professional engagement | Involving health care professionals early in a participatory design process can help develop an eHealth application and implementation strategy that aligns with stakeholders’ needs. The application was seen as a promising tool to improve access to tailored supportive care, enhance symptom awareness, promote earlier referrals, and empower patients. |
| ^14^ **Ector, 2020** | Netherlands | To develop a patient-centered innovation for patients with chronic myeloid leukemia (CML) using a design thinking methodology. | Chronic myeloid leukemia | Patients, Clinicians/Providers | Care coordination, Education/information provision, Patient-to-provider communication, Patient-to-patient communication, Financial/insurance navigation, Overall care system navigation/access to care | Web platform | Discovery, Prototyping, Testing | Interviews | Usability, User satisfaction, Navigation effectiveness | CMyLife is a multi-featured innovation created through a multidisciplinary approach with active involvement from patients. Its purpose is to equip patients with the ability to track their results, understand them, and take appropriate action. The tool also helps patients relate their results to their individual care journey. |
| ^18^ **Mendu, 2018** | USA | To develop and evaluate an interactive, culturally and linguistically tailored virtual patient educator for Hispanic women, using an iterative, user-centered design process to address health literacy challenges and improve education and counseling about cervical cancer and HPV in a rural community. | Cervical | Patients, Clinicians/Providers | Education/information provision | Mobile app | Discovery, Prototyping, Testing | Iterative design, Usability testing, Pilot testing | Patient engagement, Educational value, Feasibility and implementation logistics, Design and strategy insights | User- and human-centered design approaches, including iterative development and community-based usability testing, are essential for creating culturally and linguistically appropriate digital tools that effectively educate and engage Hispanic women about cervical cancer prevention. |
| ^19^ **Kim, 2016** | USA | To evaluates the usability of the “personal health network” (PHN), a novel solution leveraging social networking and mobile technologies, among individuals undergoing chemotherapy and receiving care coordination. | All (Adult) | Patients | Care coordination, Education/information provision, Appointment scheduling/tracking, Patient-to-provider communication, Overall care system navigation/access to care | Mobile app | Testing | Interviews, Usability testing | Usability, User satisfaction, Navigation effectiveness | Participants felt more connected to their healthcare team when using the PHN. They valued access to the patient education library and felt better able to manage the many activities involved in chemotherapy. However, improvements are still needed in navigation, connectivity, and integration with electronic health records. These findings support ongoing enhancements to the PHN and provide a roadmap for increasing its impact on technology-enabled cancer care coordination. |
| ^20^ **Mendes-Santos, 2022** | Sweden | To create iNNOV Breast Cancer (iNNOVBC), an online, personalized program based on cognitive behavioral therapy and acceptance and commitment therapy. It’s designed to help breast cancer survivors manage mild to moderate anxiety and depression, as well as improve fatigue, sleep problems, sexual health, and overall quality of life. The study also aims to assess how useful, user-friendly, and practical the iNNOVBC program is. | Breast | Patients, Clinicians/Providers | Care coordination, Education/information provision, Appointment scheduling/tracking, Patient-to-provider communication | Web platform | Prototyping | Interviews, Usability testing, Field trials, Surveys | Usability, User satisfaction, Design and strategy insights | iNNOVBC is seen as a valuable support tool by both breast cancer survivors and mental health professionals, offering a promising solution to address psychological care gaps during survivorship. To maximize their impact, such programs should be comprehensive, user-friendly, adaptable, and structured to evolve with survivors’ changing needs throughout the cancer journey. |
| ^21^ **Kuijpers, 2015** | Netherlands | To present a final design of MijnAVL (interactive portal) based on (1) health professionals' evaluation of proposed features, (2) cancer survivors’ evaluation of a first draft, and (3) cancer survivors’ evaluation of a functional online prototype. | Breast, Lung | Patients, Clinicians/Providers | Education/information provision, Appointment scheduling/tracking | Web platform | Prototyping, Testing | Interviews, Content analysis, Usability testing, Surveys | Patient engagement, Health professional engagement, Usability, User satisfaction | The iterative design of MijnAVL, an interactive portal developed with input from multiple groups of end users, resulted in a final version that was both usable and easy to understand. Usability testing showed that the website was generally simple to navigate, and its features were straightforward to use. Among the recommendations were adding a frequently asked questions section and including hyperlinks to connect different parts of the site. |
| ^22^ **Børøsund, 2018** | Norway | To develop an evidence-based electronic stress management intervention for cancer survivors. The paper details its design and development, including content creation, iterative software prototyping, and attention to security and privacy. | All (Adult) | Patients, Clinicians/Providers | Emotional/psychosocial support, Education/information provision | Mobile app | Prototyping, Testing | Interviews, Usability testing | Patient engagement, Health professional engagement, Usability, User satisfaction | Although the user-centered design process can be demanding in terms of time, effort, and cost, failing to invest adequately in this crucial phase may ultimately waste resources. This study shows how user-centered and service design methods can capture and integrate important user and stakeholder perspectives early in the design process. Combined with evidence-based principles, this approach supported the development of a stress management intervention specifically tailored for individuals living with cancer. |
| ^23^ **Ho, 2022** | USA, Africa | To assess clinicians’ perceptions of the Mobile Palliative Care Link (mPCL) application, using a user-centered design focus to evaluate its usability and utility for supporting remote symptom management in Tanzanian cancer care. | All (Adult) | Patients, Clinicians/Providers | Patient-to-provider communication, Care coordination, Overall care system navigation/access to care | Mobile app, Web platform | Testing, Implementation | Surveys, Interviews, End-user feedback sessions, System usage data | Usability, Clinical utility, User satisfaction, Navigation effectiveness | Results indicate that clinician engagement confirmed the usability and utility of mPCL as a user-centered tool for communication and symptom control, while highlighting barriers of training and infrastructure. |
| ^24^ **Ankrah, 2022** | USA | To explore the experiences of adolescent and young adult childhood cancer survivors and their caregivers during the transition to adulthood, with a focus on how social computing technologies impact the management of social, relational, and personal boundaries, to inform the user- and human-centered design of digital tools that support cancer survivorship. | All (Pediatric) | Patients, Clinicians/Providers | Emotional/psychosocial support, Education/information provision, Overall care system navigation/access to care | Conceptual design (no specific tool tested) | Discovery | Interviews, Thematic analysis | Usability, Navigation effectiveness, Design and strategy insights | Transition to adulthood for childhood cancer survivors involves complex management of multiple boundaries, which social computing technologies can affect positively or negatively. The study provides empirical insights and design recommendations to guide development of supportive digital tools for this population.    Effective digital tool design for childhood cancer survivors requires attention to their unique social and personal boundary management challenges during adulthood transition. User- and human-centered approaches are critical to creating supportive technologies that align with survivors lived experiences. |
| ^25^ **Lau, 2018** | Canada | To explore oncology nurses’ perceptions of an evidence-based e-health tool (Oncology Interactive Navigator) through a user-centered design lens, focusing on its role in supporting patient care and nursing practice. | All (Adult) | Clinicians/Providers | Education/information provision, Patient-to-provider communication, Overall care system navigation/access to care | Web platform | Discovery, Implementation | Focus groups, End-user feedback sessions | Usability, User satisfaction, Clinical utility | Conclusions suggest that incorporating nurses’ perspectives through a human-centered lens positions e-health tools like OIN as valuable for advancing practice and patient support, though integration strategies are needed. |
| ^26^ **Moradian, 2018** | Canada | To evaluate the usability a mobile phone–based technology, from the perspective of Canadian patients with cancer receiving chemotherapy to identify existing design, functionality, and usability issues and elicit their views, experiences, and satisfaction with the tool. | All (Adult) | Patients | Overall care system navigation/access to care | Web platform | Testing | Interviews, Usability testing, Surveys | Usability, User satisfaction | The ASyMS has shown positive perceptions of patients in usability testing and qualitative interviews.  Results showed that most participants were motivated to use the ASyMS, viewed it positively, and expressed interest in future use. Most identified design and functionality issues were related to the navigation of the ASyMS device and a desire for a more attractive design with advanced functionality and features. |
| ^29^ **O'Malley, 2019** | USA | To describe a user-centered development process of an eHealth tool designed to facilitate self-management of cancer survivorship follow-up care. | Breast, Prostate,  Colorectal | Patients, Clinicians/Providers | Emotional/psychosocial support, Education/information provision, Patient-to-provider communication | Web platform | Prototyping, Testing | Interviews, Usability testing | Patient engagement, Health professional engagement, Usability, User satisfaction | The top 5 functions identified in phase I for the web-based platform included: educational materials to learn and prepare for health encounters, questions for health providers, ability to track contact information of providers, provide general information and support information. Users of the prototype reported patient burden, tool fatigue, introduction timing of the tool, relevance, and security/privacy as concerns in phase II.  In general, this study demonstrates the value of using a theoretically informed and user-centered design process to develop relevant and patient-centered eHealth resources to support cancer survivorship. |
| ^30^ **Nikkhah, 2022** | USA | To understand how parents of children hospitalized for cancer adopt and manage caregiving roles during hospitalization, exploring their coordination challenges through a user-centered lens to inform the design of technologies that support multi-caregiver collaboration and role management. | All (Pediatric) | Patients, Clinicians/Providers, Caregivers | Care coordination, Emotional/psychosocial support, Overall care system navigation/access to care | Conceptual design (no specific tool tested) | Discovery | Interviews, Thematic analysis | Navigation effectiveness, Collaboration and coordination, Design and strategy insights | Applying a human-centered design approach to caregiving coordination tools—one that addresses caregivers’ lived experiences of role strain and information disparities—can improve the support provided to parents and families managing complex caregiving roles during pediatric cancer hospitalization. |
| ^31^ **Womack, 2022** | USA | To involve cancer patients directly in co-designing digital health tools and clinical processes that support better awareness and use of integrative oncology services, addressing their lived experiences and real-world needs. | All (Adult) | Patients, Clinicians/Providers | Care coordination, Education/information provision, Symptom management, Overall care system navigation/access to care | Mobile app | Discovery, Prototyping | Interviews, Participatory design sessions, Journey mapping, End-user feedback sessions | Patient engagement, Educational value, Usability, User satisfaction | Human-centered design approaches that actively engage patients in co-production lead to more relevant, usable, and meaningful digital tools and clinical processes. Collaborative design enables healthcare systems to better meet patient needs and improve integrative oncology service awareness and utilization. |
| ^32^ **Anders, 2024** | Germany | To evaluate the usability and user experience of the Enable smartphone app for breast cancer patients, incorporating patient-centered feedback to guide future design and development. | Breast | Patients | Appointment scheduling/tracking, Education/information provision, Patient-to-provider communication | Mobile app | Testing | Usability testing, Interviews, End-user feedback sessions | Usability, User satisfaction, Patient engagement | Findings suggest that patient feedback confirmed good usability of the Enable app, and that a user-centered approach points to the need for more active features and content updates. |
| ^33^ **Ahmadi, 2022** | Iran | To develop and evaluate a mobile application for breast cancer–related lymphedema self-care, using a user-centered design process based on patient needs and usability feedback. | Breast | Patients | Education/information provision, Emotional/psychosocial support, Care coordination | Mobile app | Discovery, Prototyping, Testing | Surveys, Literature/app review, Usability testing | Usability, Patient engagement, Self-management support | Findings indicate that engaging patients directly in design and usability testing led to an acceptable, user-centered mobile app for BCRL self-care; further studies should establish clinical impact. |
| ^34^ **Geerts, 2023** | Netherlands | To develop and evaluate a patient-centered, multi-modality e-health application (“MM E-coach”) designed to support patients with multiple myeloma and healthcare professionals by enhancing treatment management, usability, and communication through an iterative, user- and stakeholder-driven design process. | Multiple Myeloma | Patients, Clinicians/Providers, Caregivers | Care coordination, Education/information provision, Medication support, Patient-to-provider communication, Overall care system navigation/access to care | Mobile app | Discovery, Prototyping, Testing | Focus groups, Stakeholder consultations, Pilot study | Patient engagement, Usability, User satisfaction | The MM E-coach supports patient-centered care in multiple myeloma by enhancing treatment support for patients and providers, demonstrating promise for integration in clinical care pathways; further clinical effectiveness trials are underway. |
| ^35^ **Morse, 2021** | USA | To design, develop, and test a human-centered mobile and web app (mPCL) that supports outpatient palliative symptom assessment, care coordination, and pain control among Tanzanian cancer patients by connecting patients, caregivers, local health workers, and palliative care specialists. | All (Adult) | Patients, Clinicians/Providers, Caregivers | Care coordination, Education/information provision, Symptom management, Patient-to-provider communication, Overall care system navigation/access to care | Mobile app, Web platform | Discovery, Prototyping, Testing, Implementation | Interviews, Direct observation, Qualitative and quantitative feedback sessions | Usability, User satisfaction, Feasibility and implementation logistics, Clinical utility | The mPCL app is a usable, functional mobile solution supporting outpatient palliative care for cancer patients in Tanzania by facilitating coordinated care among patients/caregivers, local health workers, and specialists. Further studies are needed to validate its effectiveness and sustainability, especially in remote areas. |
| ^27^ **Mittal, 2025** | USA | To design, develop, and deploy SCOPE, a technology-enhanced collaborative care platform integrating a patient-facing mobile app with a provider-facing registry, aiming to improve collaboration, engagement, and psychosocial care management for cancer patients with comorbid depression through a human-centered iterative design and real-world deployment. | All (Adult) | Patients, Clinicians/Providers | Care coordination, Emotional/psychosocial support, Patient-to-provider communication, Overall care system navigation/access to care | Mobile app, Web platform | Discovery, Prototyping, Testing, Implementation | Interviews, End-user feedback sessions | Patient engagement, Navigation effectiveness, Feasibility and implementation logistics, Collaboration and coordination, Design and strategy insights | A human-centered, technology-enhanced collaborative care platform can improve engagement, communication, and coordination in managing cancer patients with depression, though real-world deployment reveals critical implementation barriers and the need for adaptable designs tailored to diverse patient needs. |
| ^36^ **Hou, 2020** | Taiwan | To investigate the information needs of Taiwanese women with breast cancer to inform the development of a Self-management support mHealth app. | Breast | Patients | Care coordination, Emotional/psychosocial support, Patient-to-provider communication, Patient-to-patient communication | Mobile app | Discovery, Prototyping | Focus groups, Interviews | Patient engagement, Usability, User satisfaction | The strengths of the design thinking approach were its focus on user-centered design and cultural sensitivity. Insights gained from each step supported the app’s development and helped bridge the gap between end users and developers. An mHealth app that incorporates eight key themes—treatment, physical activity, diet, emotional support, health records, social resources, experience sharing, and expert consultation—can better support disease self-management among Taiwanese women with breast cancer. |
| ^37^ **Schadewaldt, 2025** | Australia | To develop an evidence-informed implementation strategy for the Brain Tumors Online supportive care platform, using a user-centered approach that accounts for the needs of patients, carers, and healthcare professionals. | Brain | Patients, Caregivers, Clinicians/Providers | Education/information provision, Emotional/psychosocial support, Overall care system navigation/access to care | Web platform | Discovery | Rapid review (evidence synthesis), Stakeholder consultations | Implementation feasibility | Findings suggest that applying evidence and stakeholder input in a human-centered way informed an implementation strategy that reduces risk of digital health failure and tailors BT Online to context. |
| ^38^ **Glaser, 2025** | Netherlands | To evaluate the usability, user experience, and acceptability of the CAPABLE prototype for immunotherapy patients, emphasizing a user-centered design approach that includes both target users and broader patient groups. | Melanoma and renal cell carcinoma | Patients | Patient-to-provider communication, Education/information provision, Emotional/psychosocial support | Web platform | Testing | Interviews, Usability testing, Perceived usefulness questionnaire | Usability, User satisfaction, Patient engagement, Clinical utility | Conclusions highlight that including target users—especially immunotherapy patients—in usability testing was critical for identifying problems that a broader sample would have missed, underscoring the value of user-centered evaluation. |
| ^39^ **Baseman, 2017** | USA | To explore the feasibility and acceptability of developing a mobile health survivorship care app to facilitate care coordination; support medical, psychosocial, and practical needs; and improve survivors' long-term health outcomes. | Breast | Patients, Clinicians/Providers | Care coordination, Emotional/psychosocial support, Appointment scheduling/tracking | Mobile app | Prototyping | Usability testing, Informal questioning | Usability, User satisfaction, Navigation effectiveness | SmartSurvivor is a feasible and acceptable approach to meeting survivorship care objectives and the needs of both breast cancer survivors and their health care providers. Exploration of mobile health options for supporting survivorship care plan needs is a promising area of research. |
| ^40^ **Timmerman, 2016** | Netherlands | To report on the process of co-creation and evaluation of a multimodal ICT-supported cancer rehabilitation program with and for lung cancer patients treated with lung resection and their healthcare professionals (HCPs). | Lung | Patients, Clinicians/Providers | Education/information provision, Patient-to-provider communication, Patient-to-patient communication | Telehealth/video | Prototyping, Testing | Interviews, Usability testing, Focus groups, Scenarios | User needs and requirements, Usability, User satisfaction | A telehealth app that helps with symptom tracking and physical fitness training can support recovery after lung cancer surgery. Involving end users in the design process is important to increase the likelihood of adoption, consistent use, and successful implementation. |
| ^41^ **Iott, 2019** | USA | To investigate oncology providers’ needs and preferences regarding the design and configuration of patient-reported outcome (PRO) tools through a human-centered design approach involving prototype development, focus groups, and scenario-based feedback to enhance usability and clinical integration. | All (Adult) | Patients, Clinicians/Providers | Care coordination, Patient-to-provider communication, Overall care system navigation/access to care | Software prototype, Web platform | Discovery, Prototyping, Testing | Focus groups, Scenarios, End-user feedback sessions | Usability, Clinical utility, Feasibility and implementation logistics, Design and strategy insights | A human-centered design approach revealed oncology providers’ requirement for configurable PRO tools that offer fine-grained control, supporting their clinical workflows and improving usability in routine cancer care. |
| ^42^ **Salako, 2023** | Africa | To outline and compare current applications of remote symptom monitoring for palliative cancer care in Africa, highlighting user-centered implementation experiences to guide future development and evaluation. | All (Adult) | Patients, Clinicians/Providers | Patient-to-provider communication, Care coordination, Overall care system navigation/access to care | Web platform | Implementation | Participatory sessions, End-user feedback sessions | Navigation effectiveness, Clinical utility, Feasibility | Conclusions highlight that co-developing and adapting remote monitoring tools with local stakeholders demonstrates feasibility for palliative cancer care, with user-centered insights guiding future scale-up. |
| ^28^ **Nabelsi, 2024** | Canada | 1) To understand the interfacility thoracic surgery pathways.   2) To design, adapt, and test the platform with the target pathways; and   3) To implement the platform and evaluate the end-user experience | Lung, Esophageal | Clinicians/Providers, Patient Navigators | Care coordination, Education/information provision, Appointment scheduling/tracking, Patient-to-provider communication | Web platform | Prototyping, Testing, Implementation | Interviews, Co-design workshops, Surveys | Usability, User satisfaction, Navigation effectiveness, Clinical utility, Patient engagement, Health professional engagement | Nurse navigators are important in managing patient care. Strong leadership, teamwork among stakeholders, and smart use of technology are key to better care coordination and more efficient healthcare operations. |
| ^43^ **Aronoff-Spencer, 2022** | USA | To describe a human-centered participatory design approach that engages patients, caregivers, and community stakeholders to address a community-identified problem: supporting rural patients experiencing distress during cancer treatment | All (Adult) | Patients, Caregivers, Clinicians/Providers | Overall care system navigation/access to care | Mobile app | Discovery, Prototyping, Testing | Usability testing | Usability, User satisfaction, Patient engagement, Health professional engagement | Involving communities in defining problems and co-designing solutions through participatory, design-with methods can lead to outcomes that are more acceptable and effective compared to traditional design-for approaches. |
| ^44^ **Patel, 2024** | USA | To explore and characterize caregivers’ technology preferences and coordination needs across different phases of a child’s cancer illness and treatment using Q-methodology. | All (Pediatric) | Patients, Clinicians/Providers, Caregivers | Care coordination, Emotional/psychosocial support, Patient-to-provider communication, Overall care system navigation/access to care | Conceptual design (no specific tool tested) | Discovery | Interviews, Q-methodology | Navigation effectiveness, Design and strategy insights | Caregivers of children with cancer exhibit evolving technology preferences for caregiving coordination across illness phases, highlighting the importance of adaptable and phase-specific digital tool design. |
| ^45^ **Carrera, 2025** | Germany | To describe the user-centered development of a cancer prevention web app prototype and evaluate its usability through a graphical user interface (GUI) test. | All (Adult) | Patients | Education/information provision | Web platform | Prototyping, Testing | Interviews, Usability testing, Surveys | Usability, User satisfaction, Navigation effectiveness | The iterative development of the cancer prevention web-app provided key user insights, revealing areas for usability improvements in data entry, user guidance, and tutorials. Positive feedback on interactive features confirmed design strengths, supporting the app’s potential to enhance risk understanding and promote healthy behaviors through evidence-based communication. |
| ^46^ **Basch, 2020** | USA | To investigate the perspectives of patients, nurses, and physicians about ongoing collection of patient-reported outcomes for symptom monitoring in their community oncology clinics. | All (Adult) | Patients, Clinicians/Providers | Patient-to-provider communication | Web platform | Testing | Surveys | Usability, User satisfaction, Navigation effectiveness, Clinical utility | Most patients find online systems for reporting symptoms between visits easy to use, understandable, and helpful for communication and quality of care. Nurses and oncologists are generally enthusiastic about using patient-reported outcomes in the clinic, though some worry about the extra workload from symptom alerts. |
| ^47^ **Donawa, 2024** | USA | To evaluate the usability of open-source mobile app interfaces for rural and older cancer patients, applying a user-centered design perspective to identify barriers and opportunities for simplifying mHealth adoption. | All (Adult) | Patients | Patient-to-provider communication, Education/information provision, Overall care system navigation/access to care | Mobile app | Testing | Usability testing | Usability, User satisfaction, Identification of critical usability issues | Conclusions suggest that even without full co-design, usability testing with older and rural patients demonstrated that open-source interfaces can achieve acceptable usability when guided by human-centered heuristics. |
| ^48^ **Langius-Eklöf, 2017** | Sweden | To investigate user behavior, adherence to reporting, and the patients’ experiences of using “Interaktor” during radiotherapy for localized advanced prostate cancer. | Prostate | Patients | Overall care system navigation/access to care | Mobile app | Implementation, Testing | Interviews | User satisfaction | The use of “Interaktor” increased patients’ sense of security and their reflections on their own well-being and thereby served as a supportive tool for the self-management of symptoms during treatment of prostate cancer. |

**Full Search Strategies by Database**

1. PubMed/MEDLINE

Date of Search: July 30, 2025

Date Range: January 1, 2015 - July 30, 2025

Fields Searched: Title/Abstract

Search String:

(cancer navigation OR patient navigation OR care coordination OR care transitions OR cancer care pathway OR cancer journey support)

AND

(digital health OR mobile health OR mHealth OR m-health OR eHealth OR e-Health OR mobile application OR mobile app OR wearable OR remote monitoring OR telehealth OR tele-health OR teleoncology OR health technology)

AND

(user-centered design OR UCD OR human-centered design OR HCD OR user centered design OR human centered design OR participatory design OR (person-centered care AND design) OR usability testing)

1. Scopus

Date of Search: July 30, 2025

Date Range: January 1, 2015 - July 30, 2025

Fields Searched: Title/Abstract

Search String:

("cancer navigation" OR "patient navigation" OR "care coordination" OR "care transitions" OR "cancer care pathway" OR "cancer journey support") AND ("digital health" OR "mobile health" OR "mHealth" OR "ehealth" OR "mobile application" OR "mobile app" OR "wearable" OR "remote monitoring" OR "telehealth" OR "teleoncology") AND ("user-centered design" OR "UCD" OR "human-centered design" OR "HCD" OR "user centered design" OR "human centered design" OR "participatory design" OR ("person-centered care" AND "design") OR "usability testing")

1. IEEE Xplore

Date of Search: July 30, 2025

Date Range: January 1, 2015 - July 30, 2025

Fields Searched: Title/Abstract

Search String:

("cancer navigation" OR "patient navigation" OR "care coordination" OR "care transitions" OR "cancer care pathway" OR "cancer journey support") AND ("digital health" OR "mobile health" OR "mHealth" OR "m-health" OR "eHealth" OR "e-Health" OR "mobile application" OR "mobile app" OR "wearable" OR "remote monitoring" OR "telehealth" OR "tele-health" OR "teleoncology" OR "health technology") AND ("user-centered design" OR "UCD" OR "human-centered design" OR "HCD" OR "user centered design" OR "human centered design" OR "participatory design" OR ("person-centered care" AND "design") OR "usability testing")

1. Web of Science

Date of Search: July 30, 2025

Date Range: January 1, 2015 - July 30, 2025

Fields Searched: Title/Abstract

Search String:

TS=("cancer navigation" OR "patient navigation" OR "care coordination" OR "care transitions" OR "cancer care pathway" OR "cancer journey support") AND TS=("digital health" OR "mobile health" OR "mHealth" OR "ehealth" OR "mobile application" OR "mobile app" OR "wearable" OR "remote monitoring" OR "telehealth" OR "teleoncology") AND TS=("user-centered design" OR "UCD" OR "human-centered design" OR "HCD" OR "user centered design" OR "human centered design" OR "participatory design" OR ("person-centered care" AND "design") OR "usability testing")

1. Embase

Date of Search: July 30, 2025

Date Range: January 1, 2015 - July 30, 2025

Fields Searched: Title/Abstract

Search String:

cancer navigation AND digital health AND user-centered design

1. ACM Digital Library

Date of Search: July 30, 2025

Date Range: January 1, 2015 - July 30, 2025

Fields Searched: Title/Abstract

Search String:

("cancer navigation" OR "patient navigation" OR "care coordination" OR "care transitions" OR "cancer care pathway" OR "cancer journey support") AND ("digital health" OR "mobile health" OR mHealth OR m-health OR ehealth OR e-health OR "mobile application" OR "mobile app" OR wearable OR "remote monitoring" OR telehealth OR tele-health OR teleoncology OR "health technology") AND ("user-centered design" OR UCD OR "human-centered design" OR HCD OR "user centered design" OR "human centered design" OR "participatory design" OR ("person-centered care" AND design) OR "usability testing")

1. CINAHL

Date of Search: July 30, 2025

Date Range: January 1, 2015 - July 30, 2025

Fields Searched: Title/Abstract

Search String:

("cancer navigation" OR "patient navigation" OR "care coordination" OR "care transitions" OR "cancer care pathway" OR "cancer journey support") AND ("digital health" OR "mobile health" OR mHealth OR m-health OR ehealth OR e-health OR "mobile application" OR "mobile app" OR wearable OR "remote monitoring" OR telehealth OR tele-health OR teleoncology OR "health technology") AND ("user-centered design" OR UCD OR "human-centered design" OR HCD OR "user centered design" OR "human centered design" OR "participatory design" OR ("person-centered care" AND design) OR "usability testing")
